# Lead Poison

**Published:** 1870-02

**Authors:** 


### LEAD POISON.

Water is brought into all our city houses by pipes made out of lead, which is poisonous to the human system if it is decomposed, and water does this to a certain extent when it is allowed to remain several hours in contact with the lead. If a leaden cup were made, and it were filled with water over night and were drank in the morning, a poisoning process would commence, and would go on if the water were continued to be used every day.

Some persons would become ill and die in a few days; others would stand it out longer, but would finally be troubled with serious mysterious symptoms; a few, who had vigorous constitutions, would effectually resist the influence.

To avoid this lead poisoning, New York servants are taught to let the water flow for a few minutes every morning before it is used for cooking purposes; and servants, knowing that they run the same risk of being poisoned with their employers, do not require to be told of their duty often in this connection.

Still the evil effects of the lead are not as great as might be imagined, for chemists tell us that a kind of film or coating forms on the inner sides of these leaden pipes, which is impervious to water, and in that case, little or no harm results. Less harm would follow if all lead pipes were laid perfectly straight, or in as large curves as possible, and the workmen were required to be particular that no particles of lead or any thing else should be left within the pipe which could by any possibility retard the flow of water.

There has been an ingenious device to prevent this lead poisoning, by lining the lead pipe with block tin, which water can not corrode; but there is a practical difficulty which prevents the entire success of this plan; the block tin and lead are liable to different degrees of expansion and contraction under the influence of hot and cold water, hence the block tin is said to be liable to crack with the result of frequent breakage of the pipe, with all the expense, injury, and inconvenience incident.

The only perfect conducting material is glass; but human ingenuity has not yet been able to devise a material which will join the ends, not to say any thing of other obstacles.

But as in the largest city in the civilized world, London, the length of life is slightly greater than in the country, where there are no lead pipes,—and New York is not very far behind London in this respect,—it would seem that the injury to human health from lead-poisoning is exaggerated. Two things, however, can be done to mitigate the evil. Let the first few gallons of water drawn in our kitchens every morning be used for washing purposes, for it is wrong to let it run to waste; or turn off the water at night from the street main.

